# EEG-Based BCI Control Schemes for Lower-Limb Assistive-Robots

**DOI:** 10.3389/fnhum.2018.00312

**Published:** 2018-08-06

**Authors:** Madiha Tariq, Pavel M. Trivailo, Milan Simic

**Affiliations:** School of Engineering, RMIT University Melbourne, Melbourne, VIC, Australia

**Keywords:** brain-computer interface (BCI), electroencephalography (EEG), spinal cord injury (SCI), exoskeletons, orthosis, assistive-robot devices

## Abstract

Over recent years, brain-computer interface (BCI) has emerged as an alternative communication system between the human brain and an output device. Deciphered intents, after detecting electrical signals from the human scalp, are translated into control commands used to operate external devices, computer displays and virtual objects in the real-time. BCI provides an augmentative communication by creating a muscle-free channel between the brain and the output devices, primarily for subjects having neuromotor disorders, or trauma to nervous system, notably spinal cord injuries (SCI), and subjects with unaffected sensorimotor functions but disarticulated or amputated residual limbs. This review identifies the potentials of electroencephalography (EEG) based BCI applications for locomotion and mobility rehabilitation. Patients could benefit from its advancements such as wearable lower-limb (LL) exoskeletons, orthosis, prosthesis, wheelchairs, and assistive-robot devices. The EEG communication signals employed by the aforementioned applications that also provide feasibility for future development in the field are sensorimotor rhythms (SMR), event-related potentials (ERP) and visual evoked potentials (VEP). The review is an effort to progress the development of user's mental task related to LL for BCI reliability and confidence measures. As a novel contribution, the reviewed BCI control paradigms for wearable LL and assistive-robots are presented by a general control framework fitting in hierarchical layers. It reflects informatic interactions, between the user, the BCI operator, the shared controller, the robotic device and the environment. Each sub layer of the BCI operator is discussed in detail, highlighting the feature extraction, classification and execution methods employed by the various systems. All applications' key features and their interaction with the environment are reviewed for the EEG-based activity mode recognition, and presented in form of a table. It is suggested to structure EEG-BCI controlled LL assistive devices within the presented framework, for future generation of intent-based multifunctional controllers. Despite the development of controllers, for BCI-based wearable or assistive devices that can seamlessly integrate user intent, practical challenges associated with such systems exist and have been discerned, which can be constructive for future developments in the field.

## Introduction

The field of assistive technologies, for mobility rehabilitation, is ameliorating by the introduction of electrophysiological signals to control these devices. The system runs independent of physical, or muscular interventions, using brain signals that reflect user's intent to control devices/limbs (Millán et al., 2010; Lebedev and Nicolelis, 2017), called brain-computer interface (BCI). Commonly used non-invasive modality to record brain signals is electroencephalography (EEG). EEG signals are deciphered to control commands in order to restore communication between the brain and the output device when the natural communication channel i.e., neuronal activity is disrupted. Recent reviews on EEG-BCI for communication and rehabilitation of lower-limbs (LL) could be found in (Cervera et al., 2018; Deng et al., 2018; He et al., 2018a; Lazarou et al., 2018; Semprini et al., 2018; Slutzky, 2018).

About five decades ago, EEG-BCIs used computer cursor movements to communicate user intents for patient-assistance in various applications (Vidal, 1973; Wolpaw et al., 2002; Lebedev and Nicolelis, 2017). The applications are now widespread, as machine learning has become one essential component of BCI, functional in different fields of neurorobotics and neuroprosthesis. For lower extremity, applications include human locomotion assistance, gait rehabilitation, and enhancement of physical abilities of able-bodied humans (Deng et al., 2018). Devices for locomotion, or mobility assistance, vary from wearable to (non-wearable) assistive-robot devices. Wearable devices such as exoskeletons, orthosis, prosthesis, and assistive-robot devices including wheelchairs, guiding humanoids, telepresence and mobile robots for navigation are the focus of our investigation.

Control schemes, offered by these systems, rely on the inputs derived from electrophysiological signals, electromechanical sensors from the device, and the deployment of finite state controller that attempts to implicate user's motion intention, to generate correct walking trajectories with wearable robots (Duvinage et al., 2012; Jimenez-Fabian and Verlinden, 2012; Herr et al., 2013; Contreras-Vidal et al., 2016). Input signals are typically extracted from the residual limb/muscles i.e., amputated or disarticulated lower-limbs (LL), via electromyography (EMG), from users with no cortical lesion or intact cognitive functions. Such solutions consequently preclude patient groups whose injuries necessitate direct cortical input to the BCI controller, for instance users with neuromotor disorders such as spinal cord injury (SCI) and stroke, or inactive efferent nerves/synergistic muscle groups. In this case direct cortical inputs from EEG could be the central-pattern-generators (CPG) that generate basic motor patterns at the supraspinal or cortical level (premotor and motor cortex); or the LL kinesthetic motor imagery (KMI) signals (Malouin and Richards, 2010). The realization of BCI controllers solely driven by EEG signals, for controlling LL wearable/assistive devices, is therefore possible (Lee et al., 2017). Several investigations reinstate that CPG with less supraspinal control is involved in the control of bipedal locomotion (Dimitrijevic et al., 1998; Beloozerova et al., 2003; Tucker et al., 2015). This provides the basis for the development of controllers, directly driven from cortical activity in correlation to the user intent for volitional movements (Nicolas-Alonso and Gomez-Gil, 2012; Angeli et al., 2014; Tucker et al., 2015; Lebedev and Nicolelis, 2017) instead of EMG signals. Consequently, controllers with EEG-based activity mode recognition for portable assistive devices, have become an alternative to get seamless results (Presacco et al., 2011b). However, when employing EEG signals as input to the BCI controller, there necessitates a validation about the notion that EEG signals from the cortex can be useful for the locomotion control.

Though cortical sites encode movement intents, the kinetic and kinematic changes necessary to execute the intended movement, are essential factors to be considered. Studies indicate that the selective recruitment of embedded “muscle synergies” provide an efficient means of intent-driven, selective movement, i.e., these synergies, stored as CPGs, specify spatial organization of muscle activation and characterize different biomechanical subtasks (Chvatal et al., 2011; Chvatal and Ting, 2013). According to Maguire et al. (2018), during human walking, Chvatal and Ting (2012) identified different muscle synergies for the control of muscle activity and coordination. According to Petersen et al. (2012), the swing-phase was more influenced by the central cortical control, i.e., dorsiflexion in early stance at heel strike, and during pre-swing and swing phases for energy transfer from trunk to leg. They also emphasized the importance of cortical activity during steady unperturbed gait for the support of CPG activity. Descending cortical signals communicate with spinal networks to ensure that accurate changes in limb movement have appropriately integrated into the gait pattern (Armstrong, 1988). The subpopulations of motor-cortical neurons activate sequentially amid the step cycle particularly during the initiation of pre-swing and swing (Drew et al., 2008). The importance of cortical activation upon motor imagery (MI) of locomotor tasks has been reported in Malouin et al. (2003) and Pfurtscheller et al. (2006b). Similarly, the confirmation of electrocortical activity coupled to gait cycle, during treadmill walking or LL control, for applications as EEG-BCI exoskeletons and orthotic devices, has been discerned by (He et al., 2018b, Gwin et al. (2010, 2011), Wieser et al. (2010), Presacco et al. (2011a), Presacco et al. (2011b), Chéron et al. (2012), Bulea et al. (2013), Bulea et al. (2015), Jain et al. (2013), Petrofsky and Khowailed (2014), Kumar et al. (2015), and Liu et al. (2015). This provides the rationale for BCI controllers that incorporate cortical signals for high-level commands, based on user intent to walk/bipedal locomotion or kinesthetic motor imagery of LL.

**Figure 1:**
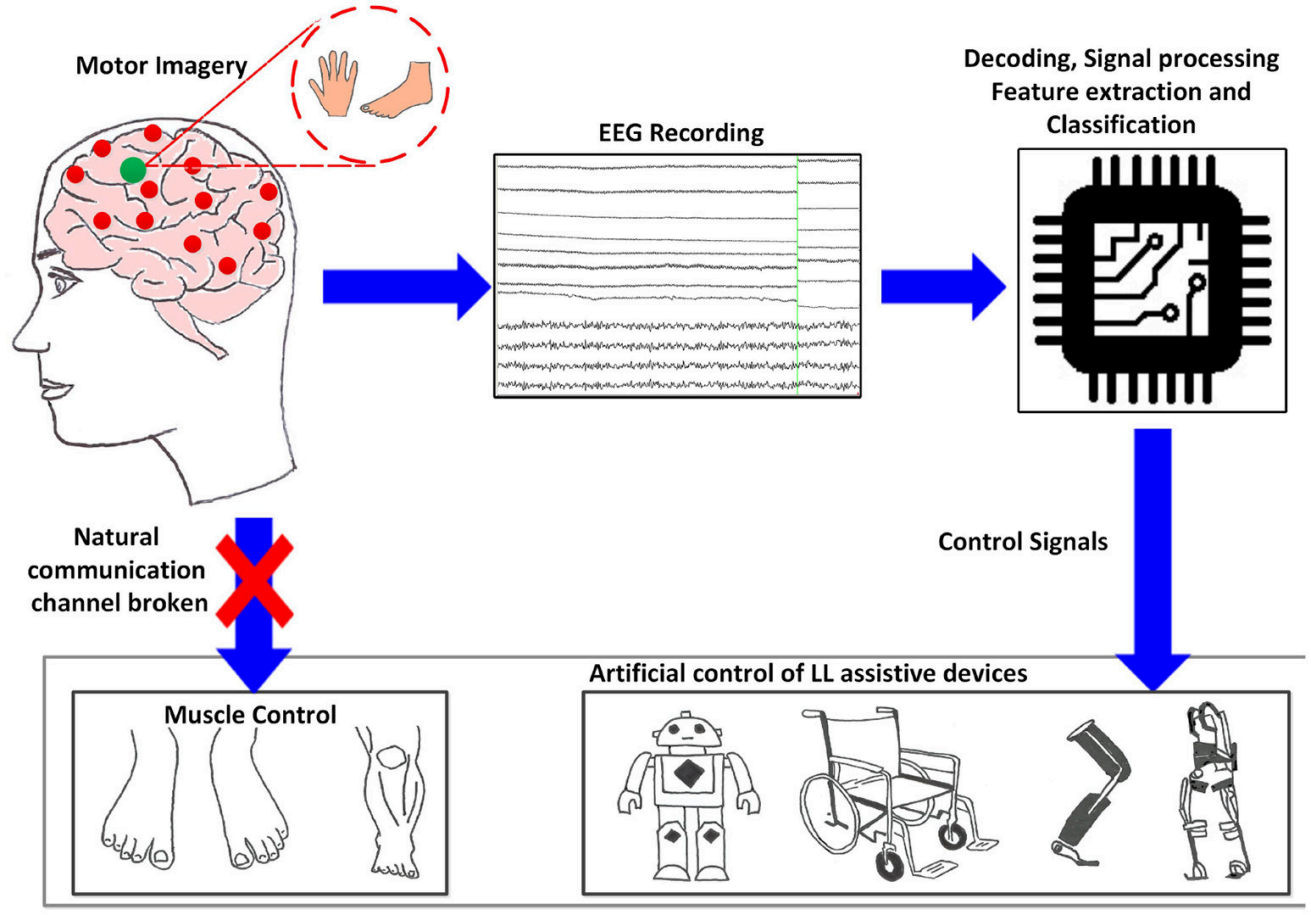
Generic concept/function diagram of BCI controlled assistive LL devices based on motor imagery.

While BCIs may not require any voluntary muscle control, they are certainly dependent on brain response functions therefore the choice of BCI depends on the user's sensorimotor lesion and adaptability. Non-invasive types of BCI depend on EEG signals used for communication, which elicit under specific experimental protocols. Deployed electrophysiological signals that we investigate, include oscillatory/sensorimotor rhythms (SMR), elicited upon walking intent, MI or motor execution (ME) of a task, and evoked potentials as event-related potentials (ERP/P300) and visual evoked potentials (VEP). Such BCI functions as a bridge to bring sensory input into the brain, bypassing damages sight, listening or sensing abilities. Figure 1 shows a schematic description of a BCI system based on MI, adapted from He et al. (2015). The user performs MI of limb(s), which is encoded in EEG reading; features representing the task are deciphered, processed and translated to commands in order to control assistive-robot device.

Reviewed control schemes deployed by wearable LL and assistive-robots are presented in a novel way, i.e., in form of a general control framework fitting in hierarchical layers. It shows the informatic interactions, between the user, the BCI operator, the shared controller, and the robot device with environment. The BCI operator is discussed in detail in the light of the feature extraction, classification and execution methods employed by all reviewed systems. Key features of present state-of-the-art EEG-based BCI applications and its interaction with the environment are presented and summarized in the form of a table. Proposed BCI control framework can cater similar systems based on fundamentally different classes. We expect a progress in the incorporation of the novel framework for the improvement of user-machine adaptation algorithms in a BCI.

The reviewed control schemes indicated that the MI/ME of LL tasks, as aspects of SMR-based BCI have not been extensively used compared to upper limbs (Tariq et al., 2017a,b, 2018). This is due to the small representation area of LL, in contrast to upper limbs, located inside the interhemispheric fissure of the sensorimotor cortex (Penfield and Boldrey, 1937). The review is an effort to progress the development of user's mental task related to LL for BCI reliability and confidence measures.

Challenges presently faced by EEG-BCI controlled wearable and assistive technology, for seamless control in real-time, to regain natural gait cycle followed by a minimal probability of non-volitional commands, and possible future developments in these applications, are discussed in the last section.

## General control framework for BCI wearable lower-limb and assistive-robot devices

**Figure 2:**
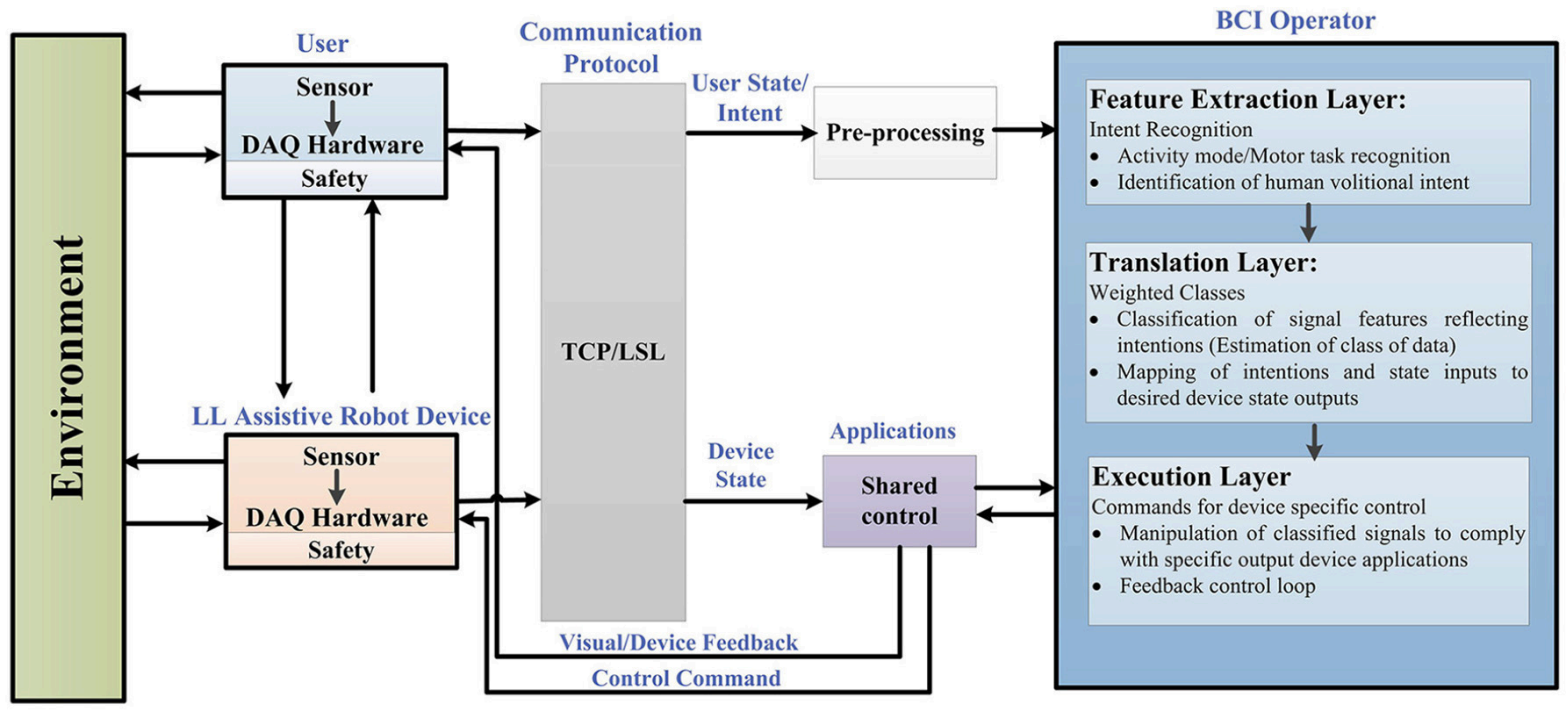
Generalized framework in BCI controlled wearable LL and assistive devices for rehabilitation.

In order to structure the control architecture adopted by various BCI wearable LL and assistive robot-devices, a general framework is presented in Figure 2. This framework was extended from Tucker et al. (2015) applicable to a range of EEG-BCI controlled devices for LL assistance, including portable exoskeletons, orthosis, prosthesis, and assistive-robots (wheelchairs, humanoids, and navigation/telepresence robots).

Figure 2 reflects the generalized control framework, where electrophysiological and transduced signal interactions, along the feedforward and feedback loops, are shown for motion intent recognition, during activity mode. Integral parts of the framework include a user of the assistive robot-device, the assistive-robot device itself, a BCI operator structure with sub-level controls, shared control, communication protocol and the interaction with environment. The BCI operator structure constitutes of three sub-layers which are the feature extraction, translation and execution layer, respectively. As a precaution to ensure human-robot interaction safety, safety layers are used with the user and the robotic device parts of the framework. The control framework is in a generalized form applicable to all brain-controlled assistive robots.

BCI control is driven from the recognition of user's motion intentions; therefore we begin from the point of origin where motion intentions arise (cortical levels). The first step involves how to perceive and interpret the user's physiological state (i.e., MI/ME or ERP) acquired via EEG. Following this, the status of physical interaction between the user and the environment (and vice versa), and the robotic device and the environment (and vice versa) are checked. The assistive-robot's state is determined via electromechanical sensors. The user and assistive-robot status inputs to the BCI operator and shared controller, respectively.

Raw signals from the user and assistive LL device pass through the communication protocol which directs them to the connected client i.e., BCI operator via pre-processing and shared control module. Real-time signal acquisition and operating software could be used to assign event markers to the recorded data e.g., OpenViBE, BioSig, BCI++, BCI2000 etc. (Schalk et al., 2004; Mellinger and Schalk, 2007; Renard et al., 2010). The streaming connection can be made using TCP (when the time synchronization requirements do not need accuracy <100 ms) or LSL which incorporates built-in network and synchronization capabilities (with accuracy of 1 ms) recommended for applications based on ERPs.

Under the control framework components, BCI operator is the core part comprising of three sub layers, described in detail in section BCI Operator.

At feature extraction layer (intent recognition), user's intent of activities related to LL movements are perceived, discerned and interpreted. Signal features associated to user's kinesthetic intent/execution of motor task (in case of SMR) are encoded in form of feature vector (Lotte, 2014). The activity-mode recognition for ERP, against displayed oddball menu for specific location, uses frequency, or time domain features. It is the user's direct volitional control that lets voluntarily manipulate the state of the device (e.g., joint position, speed, velocity and torque).

Translation layer (weighted class) takes account of the translation of extracted signal features to manipulate the robotic device, via machine understandable commands, which carry the user's intent. This is done by supervised, or unsupervised learning (classification algorithm) which essentially estimates the weighted class, represented by the feature vector, and identifies the cognitive patterns for mapping to the desired state (unique command).

The desired state of user intent is carried to the execution layer (commands for device-specific control) where an error approximation is done with reference to current state. The state of the device is also sent to the execution layer via shared controller, as a feedforward control, in order to comply with the execution layer. The execution layer sends control commands to the actuator(s) of the device and visual feedback to the user via shared control unit in order to minimize the possible error. The feedback control plays a vital role in achieving the required output (usually accounts for the kinematic or kinetic properties of the robot-device).

This closes the overall control loop and the robotic device actuates to perform the required task(s). As the wearable assistive-robot is physically placed in close contact with the user, and that the powered device is likely to generate output force, safety mechanisms are kept into consideration with the user and hardware in the control framework. Inter-networking between subsystems of the generalized control architecture relies on the exchange of information sent at signal-level as well as physical-level.

## User adaptability and EEG signal acquisition

The type of BCI is directed based on the user's lesion level and extent of adaptability to adhere with the specific BCI protocol.

### User adaptability

In order for the portable LL wearable-BCI controllers to be compliant with residual neuromusculoskeletal structures, the sensorimotor control loop of human locomotion is taken into account, since the volitional and reflex-dependent modulation of these locomotion patterns emerges at the cortical levels (Armstrong, 1988; Kautz and Patten, 2005; Bakker et al., 2007; Zelenin et al., 2011; Pons et al., 2013; Angeli et al., 2014; Marlinski and Beloozerova, 2014; Capogrosso et al., 2016). This may essentially preclude the direct control of LL via neural activity alone, while keeping a balance and orientation during dynamic tasks. However, the sole employment of cortical activity is still useful for providing high-level commands to the controller of the device to execute volitional movements (Carlson and Millan, 2013; Contreras-Vidal and Grossman, 2013; Kilicarslan et al., 2013), for patients whose injuries necessitate a direct input from cortex to the robotic device controller. Therefore, the critical aspect for a functional portable LL device is the lesion measure and the physiological constraints based on which the user can adapt to the BCI protocol. The physiological constraints in such cases can be compensated through assistance, like shared control.

### EEG signal acquisition

The neuronal activity can be divided into spikes and field potentials. Spikes show action potentials of neurons individually and are detected via invasive microelectrodes. Field potentials on the other hand can be measured by EEG and they reflect the combined synaptic, axonal and neuronal activity of the neuron groups (Yang et al., 2014; He, 2016).

The communication components in EEG activity useful for BCI include, the oscillatory activity comprising of delta, theta, alpha/mu, beta and gamma rhythms; the ERP (P300), the VEP, and slow cortical potentials (SCP). Oscillatory rhythms fluctuate according to the states of brain activity; some rhythms are distinguished depending on these states (Semmlow and Griffel, 2014). The *Mu* and *beta* rhythms are also termed SMR. The SMR elicit event-related desynchronization (ERD) or event-related synchronization (ERS) which are directly related to proportional power decrease upon ME/MI of limb(s) movement or power increase in the signal upon rest, respectively; they are non-phase locked signals (Kalcher and Pfurtscheller, 1995). Evoked potentials on the other hand are phase-locked. A BCI system employs evoked potentials when requiring less or no training from the user i.e., a system based on stimulus-evoked EEG signals that provides task-relevant information (Baykara et al., 2016), useful for locked-in or multiple sclerosis patients. This involves the presentation of an odd-ball paradigm in case of P300 or multiple visual stimuli flashing, e.g., letters, digits on screen in case of VEP. The P300 is derived from user response that evokes approximately 300 ms after stimulus triggering and corresponds to positive voltage peak (Lazarou et al., 2018). VEP measures the time for the visual stimulus to travel from the eye to occipital cortex.

**Figure 3:**
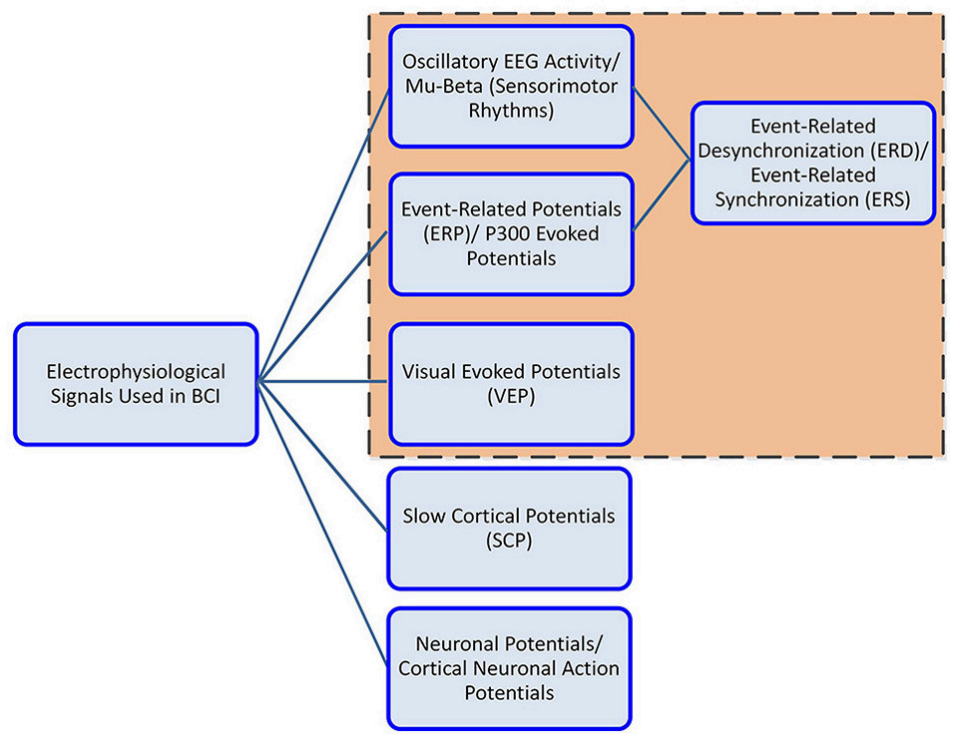
Electrophysiological signals used in BCI controlled wearable LL and assistive-robot devices.

Users can generally be grouped based on their physical and mental state, for instance locked-in patients with intact eye muscles, can communicate via ERP signals, whereas patients with motor complete but sensory incomplete SCI can utilize SMR signals based on MI. Figure 3 shows the electrophysiological signals that are extensively employed by BCI system for communication; however EEG signals employed by the wearable LL and assistive devices are highlighted for this study.

#### Deployed oscillatory rhythms

For assistive devices, the two commonly used SMR acquired from the motor cortex are *mu* (8–11 Hz) and *beta* (12–30 Hz) rhythms, which elicit upon ME/MI tasks. The ME task is based on the physical motion of the user's limbs that activate the motor cortex; this includes the development of muscular tension, contraction or flexion. The MI is a covert cognitive process based on the kinesthetic imagination of the user's own limb movement with no muscular activity also termed “kinesthetic motor imagery” (KMI) (Mokienko et al., 2013). Motor tasks can generally be upper or lower limb related (Malouin et al., 2008). The upper limb motor tasks activate hand area (Vasilyev et al., 2017) and LL motor tasks activate foot representation area of the cortex respectively (Wolpaw and Wolpaw, 2012). The advantage with MI signals is that they are free of proprioceptive feedback unlike ME tasks.

It was suggested by Wolpaw and Mcfarland (2004), that the use of *mu* and *beta* rhythms could give similar results as those presented by invasive methods for motor substitution. A non-invasive BCI could clinically support medical device applications (as discussed in section Lower-Limb Assistive-Robot Applications in Different Environments). The BCIs for control of medical device applications are reported in Allison et al. (2007); Daly and Wolpaw (2008), and Frolov et al. (2017). It was observed that BCI employed by assistive-robot devices for control purposes was focused on upper limb MI (Belda-Lois et al., 2011) such as hand and fingers, for applications including BCI hand orthotics and exoskeleton (Schwartz et al., 2006; Soekadar et al., 2015). This is because the foot representation area is near the mantelkante, which is situated deep within interhemispheric fissure of the human sensorimotor cortex (Penfield and Boldrey, 1937). However, it never withheld progress into this direction. Research on LL, precisely the foot MI/ME for controlling assistive robots, is in progress (Pfurtscheller et al., 2006a; Hashimoto and Ushiba, 2013; Tariq et al., 2017b, 2018). It was proved that the induction of *beta* ERS in addition to *mu-beta* ERD, improved the discrimination between left and right foot imagery and stepping tasks, as accurate as hand MI (Pfurtscheller et al., 2005, 2006a; Pfurtscheller and Solis-Escalante, 2009; Hashimoto and Ushiba, 2013; Liu et al., 2018) which provides a basis for research in BCI controlled foot neuroprosthesis. To our knowledge no literature on explicit employment of knee or hip KMI tasks in any BCI experimental protocol is available except for (Tariq et al., 2017a).

Besides the KMI of LL, cortical signals arising from the sensorimotor control loop of human locomotion intent is taken into account, for the portable LL wearable-BCI controllers to be compliant with the residual neuromusculoskeletal structures (La Fougere et al., 2010) suggested that brain areas underlying walking MI overlie the supplementary motor area and pre-frontal cortex. The idea of walking from thought based on foot imagery has also been presented in Pfurtscheller et al. (2006b). A novel way of therapy that earlier provided limited grade of motor-function recovery for chronic gait function impaired subjects due to foot-drop was described (Do et al., 2011, 2012). They integrated EEG-based BCI with non-invasive functional electrical stimulation (FES) system. It resulted in enabling the brain-control of foot dorsiflexion directly in healthy individuals. Takahashi et al. (2009, 2012) validated the feasibility of short-term training by employing ERD and FES based on dorsiflexion of paralyzed ankle experiments. Beta corticomuscular coherence (CMC) gave a measure of communication amid sensorimotor cortex and muscles. García-Cossio et al. (2015) demonstrated the possibility to decode walking intentions from cortical patterns. Raethjen et al. (2008) found coherence in EEG at stepping frequency and electromyography (EMG) anterior tibial muscles pattern for rhythmic foot movements.

Work on analyzing EEG signals for detection of unexpected obstacles during walking was presented recently (Salazar-Varas et al., 2015). Observation of electrocortical activity related to walking gait-cycle and balancing experiments has been reported in Presacco et al. (2011b). Electrocortical activity resulting from gait-like movements and balancing with treadmill, Erigo R tilt table, and customized stationary bicycle with rigid reclined backboard (as pedaling device) have been discussed in Wieser et al. (2010), Gwin et al. (2011), Presacco et al. (2011a), Jain et al. (2013), Petrofsky and Khowailed (2014), Bulea et al. (2015), Kumar et al. (2015), and Liu et al. (2015).

#### Deployed event-related and evoked potentials

ERPs have successfully been deployed in ambulatory and motor conditions without affecting the recorded EEG data. P300 showed to improve the performance of an EEG-based BCI system during ambulatory conditions or foot dorsiflexion/plantar-flexion condition (Lotte et al., 2009; Castermans et al., 2011b; Duvinage et al., 2012). They used similar experimental protocol i.e., oddball paradigm while subjects were physically walking or moving feet in dorsiflexion or plantar-flexion direction. In addition to this, the somatosensory evoked potentials (SEP) were deployed in assistive technologies. These potentials commonly elicit by bipolar transcutaneous electrical stimulation applied on the skin over the trajectory of peripheral nerves of the upper limb (the median nerve) or LL (the posterior tibial nerve), and then recorded from the scalp (Sczesny-Kaiser et al., 2015). In addition to the wearable devices, assistive technologies as EEG-BCI controlled wheelchairs and humanoid robots have successfully deployed the P300 (Rebsamen et al., 2007, 2010; Pires et al., 2008; Iturrate et al., 2009b; Palankar et al., 2009; Lopes et al., 2011; Kaufmann et al., 2014) and VEP signals (Bell et al., 2008). However, the only drawback, with employment of ERP and VEP signals in a BCI for the control of assistive devices precisely wearables, is the presence of visual stimulus set-up within the device that makes it less convenient for portable applications.

## Communication protocol

Like a basic communication system, the BCI for control of assistive devices has an input, an output, translation components for converting input to output, and a protocol responsible for the real-time operation onset, offset and timing.

Acquired EEG signals are transferred to the BCI operator via a communication protocol. Similarly sensor output from the robot device is directed to the shared control unit via communication protocol, Figure 2. Communication protocol could be a transmission control/internet protocol (TCP/IP), a suite of communication protocols used to interconnect network devices on the internet or a private network. For instance, in EEG-BCI controlled humanoids, the data (visual feedback images from the humanoid monocular camera and motion commands from the BCI system) were transmitted using wireless TCP/IP communication between the humanoid and other systems (Chae et al., 2011a,b, 2012).

An alternate approach is the lab streaming layer (LSL), which allows synchronization of the streaming data across devices. Information can be streamed over the network from “Presentation to the LSL” (Iturrate et al., 2009b; Renard et al., 2010; Kothe and Makeig, 2013; Gramann et al., 2014). Recent assistive applications (Galán et al., 2008; Millán et al., 2009) such as wheelchairs, and mobile robots, use controller area network (CAN) bus which is a robust vehicle bus standard. It is designed to allow microcontrollers and devices to communicate in applications without a host computer and follows a message-based protocol. It is a low cost, fault tolerant communication system, with the data transfer rates in the range of 40 Kbit/s to 1 Mbit/s.

## BCI operator

After passing through the communication protocol, acquired EEG signals are directed to connected client, i.e., the BCI operator, but are pre-processed first.

### Preprocessing

The acquired raw EEG signals are pre-processed, as they are susceptible to noise and artifacts. It could be hardware/environmental noise, experimental error or physiological artifact. As hardware and environmental noise are not brain-related, it is best to remove them before converting raw EEG to signal features.

#### Removal of noise

Hardware noise in the EEG signal usually occurs due to instrument degradation, electrode wear, mains interference (AC power lines), electromagnetic wave sources as computers, mobile phones, notebooks, wireless routers or other electronic equipment. High noise frequencies in the signal can be removed by notch filters (50 or 60 Hz for power lines). To block electromagnetic waves, electromagnetic shields could be used.

#### Removal of artifacts

EEG artifacts arise due to physiological activities such as skin impedance fluctuations, electrooculography activity, eye blinks, electrocardiographic activity, facial/body muscle EMG activity and respiration. As the frequency ranges, for the aforementioned physiological signals are typically known, the bandpass filter can be an effective preprocessing tool. Most EEG-based BCI systems for assistive technologies have shown the successful implementation of simple low-pass, high-pass, or bandpass filters to remove physiological artifacts. Other methods for artifact removal include temporal filtering, spatial filtering, independent component analysis (ICA) (Viola et al., 2009), principal component analysis (PCA), linear regression, blind source separation (BSS) (Ferdousy et al., 2010), wavelet transform, autoregressive moving average, nonlinear adaptive filtering, source dipole analysis (Fatourechi et al., 2007) or thresholding of meaningful parameters (e.g., channel variance) based on a prior statistical analysis (Nolan et al., 2010).

### Feature extraction layer

After preprocessing of data, different brain activities are classified based on their selected features.

#### Band power features

The band power features, usually used, are the time-frequency components of ERD/ERS. After bandpass filtering, resulting signal is squared to obtain its power *p*[*t*] = *x*^2^[*t*], where *x* is the filtered single band EEG signal amplitudes and *p* is the resulting band-power values. To smooth-out (average) the signal, a *w*-sized smoothing window operation is used. This is followed by a logarithm of the processed signal sample, using Equation 1:

(1) $$\begin{array}{r} \bar{p}\left[n\right]=\ln\left(\frac{1}{w}\overset{w}{\underset{k=0}{\sum }}p\left[n-k\right]\right) \end{array}$$

where $\bar{p}\left[n\right]$ are the smoothed band-power values, and *w* is the smoothing window size. In their work (Presacco et al., 2011b; Contreras-Vidal and Grossman, 2013), the feature extraction method employed by EEG-BCI lower exoskeleton, for neural decoding of walking pattern, included power spectral density (PSD) analysis of the kinematic data and adaptive Thompson's multitaper for each channel of EEG recorded, during rest and walking tasks. Decoding method employed a time-embedded linear Wiener filter, independently designed and cross-validated for each extracted gait pattern. Parameters of the model were calculated with Gaussian distribution method. This ensured the feasibility of successfully decoding human gait patterns with EEG-BCI LL exoskeleton. Similarly, the results tested a on paraplegic subject for BCI controlled lower exoskeleton (Kilicarslan et al., 2013) reflect the method of decoding closed loop implementation structure of user intent with evaluation accuracy of 98%. Data was filtered in *delta* band (0.1–2 Hz) using 2nd order Butterworth filter. The filtered data was standardized and separate channels were used, to create feature matrix to extract *delta* band features.

In 2012 (Noda et al., 2012) proposed an exoskeleton robot that could assist user stand-up movements. For online decoding they used 9th order Butterworth filter for 7–30 Hz band. After down-sampling, Laplace filter and common average subtraction were applied for voltage bias removal. The covariance matrix of the processed data was used as input variable for the two-class classifier; the results were productive. Other EEG-BCI lower exoskeletons (Gancet et al., 2011, 2012) considered employing steady-state VEP (SSVEP) for motion intention recognition. Proprioceptive artifacts removal (during walk) is aimed to be removed using ICA. Other recent work on LL exoskeleton controlled via SSVEP includes (Kwak et al., 2015). In the SEP-controlled LL exoskeleton (Sczesny-Kaiser et al., 2015), SEP signals were sampled at 5 kHz and bandpass filtered between 2 and 1,000 Hz. In total 800 evoked potentials were recorded in epochs from 30 before to 150 ms after the stimulus, and then averaged. Paired-pulse suppression was expressed as a ratio of the amplitudes of second and first peaks, which was the primary outcome parameter. For correlation analysis, they calculated the difference of mean amplitude ratios.

For a BCI controlled robotic gait orthosis (Do et al., 2011, 2013) an EEG prediction model was generated to exclude EEG channels with excessive artifacts. The EEG epochs corresponding to idling and walking states were then transformed into frequency domain, their PSD were integrated over 2 Hz bins, followed by dimensionality reduction using class-wise principal component analysis (CPCA). The results established feasibility of the application.

BCI and shared control wheelchairs, based on MI signals to ensure interference free navigation protocol, was presented in Millán et al. (2009) and Carlson and Millan (2013). They estimated PSD in the 4–48 Hz band with a 2 Hz resolution. ERD was observed in the *mu* band power 8–13 Hz. These changes were detected by estimating the PSD features every 16 times/s using Welch method with five overlapped (25%) Hanning windows of 500 ms. In order to select subject-specific features, that maximize the separability between different tasks (based on training data cross validation) the canonical variate analysis (CVA) was used. In a similar work presented by Galán et al. (2008) for BCI controlled wheelchair, feature selection was done by picking stable frequency components. The stability of frequency components was assessed using CVA one per frequency component on the training set.

#### Time-domain parameters

The time-domain parameters compute time-varying power of the first *k* derivatives of the signal; $p_{i}\left(t\right)=\frac{d^{i}x\left(t\right)}{dt^{i}}$ where *i* = 0, 1, …, *k* and *x* is the initial EEG signal. Resulting derivatives are smoothed using exponential moving average and logarithm, used in feature vector generation, as given in Equation 2:

(2) $$\begin{array}{r} \bar{p_{i}}\left[n\right]=\ln\left(up_{i}\left[n\right]-\left(1-u\right)p_{i}\left[n-1\right]\right) \end{array}$$

where $\bar{p}$ is the smoothed signal derivatives, *u* is the moving average parameter, *u*∈[0;1].

EEG-BCI for control of LL orthosis (Taylor et al., 2001; Duvinage et al., 2012) combined a human gait model based on a CPG and a classic but virtual P300 to decipher user's intent for four different speeds. P300 was used to control the CPG model and the orthosis device by sending high-level commands. The frequency band for P300 were high-pass filtered (temporal) at 1 Hz cut off frequency using 4th order Butterworth filter. This was followed by designing of an xDAWN-based spatial filter, by linearly combining EEG channels. When EEG signals were projected into this subspace, P300 detection was enhanced. The resulting signal was epoched using time window that started after stimulus, averaged and sent to the classifier. In another related work (Lotte et al., 2009), the epoching of P300 signal was done by selection of related time window, followed by bandpass filtering in 1–12 Hz range using 4th order Butterworth filter. Post this; winsorizing for each channel was done by replacing values within 5% most extreme values by most extreme values from remaining 95% samples from that window. A subset of the features was selected using the sequential forward floating (SFFS) feature selection algorithm that ensured the maximization of performance of the BCI system.

The EEG-BCI for foot orthosis reported in Xu et al. (2014), employed bandpass filtering (0–3 Hz). The system was based on the detection of movement-related cortical potentials (MRCP). The data between 0.5 and before 1.5 s, after the movements, were extracted as the “signal intervals” while others were extracted as the “noise intervals.” The measure analysis of variance, ANOVA, was used for statistical analysis.

The P300-BCI wheelchair incorporated bandpass filtering between 0.5 and 30 Hz and characterized the P300 signal in the time domain. For each EEG channel, 1-s sample recordings were extracted after each stimulus onset and filtered using the moving average technique. The resulting data segments for each channel selected were concatenated, creating a single-feature vector (Iturrate et al., 2009a,b).

#### Common spatial patterns

The common spatial pattern (CSP) features are sourced from a preprocessing technique (filter) used to separate a multivariate signal into subcomponents that have maximum differences in variance (Müller-Gerking et al., 1999). The difference allows simple signal classification. Generally, the filter can be described as a spatial coefficient matrix *W*, as shown in Equation 3:

(3) $$\begin{array}{r} S=W^{T}E \end{array}$$

where *S* is the filtered signal matrix, *E* is the original EEG signal vector. Columns of *W* denote spatial filters, while *W*^T^ are the spatial patterns of EEG signal. In their work (Choi and Cichocki, 2008) used SMR to control wheelchair. For pre-processing they employed the second order BSS algorithm using a modified and improved real-time AMUSE algorithm that enabled a rapid and reliable estimation of independent components with automatic ranking (sorting) according to their increasing frequency contents and/or decreased linear predictability. The AMUSE algorithm worked as 2 consecutive PCAs; one applied to the input data and the second applied to the time-delayed covariance matrix of the output from the previous stage. For feature extraction, CSP filter was used that distinguish each data group optimally from the multichannel EEG signals.

SMR-based humanoid robots used the KMI of left hand, right hand, and foot as control signals (Chae et al., 2011b, 2012). Sampled EEG signals were spatially filtered with large Laplacian filter. During the overall BCI protocols, Laplacian waveforms were subjected to an autoregressive spectral analysis. For amplitude features extraction, every 250 ms observation segment was analyzed by the autoregressive algorithm, and the square root of power in 1 Hz wide frequency bands within 4–36 Hz was calculated.

### Translation layer

After passing through the feature extraction layer, the feature vector is directed to the translation layer to identify user intent brain signals, and manipulate the robotic device via machine understandable commands for interfacing. Different classification techniques for distinct features are used. Classification algorithms, calibrated via supervised or unsupervised learning, during training phase, are able to detect brain-signal patterns during the testing stage. This essentially estimates the weighted class, represented by the feature vector for mapping to the desired state (unique command). A recent review on most commonly used classification algorithms for EEG-BCIs has been reported by (Lotte et al., 2018). Some of the commonly used classification methods in EEG-BCI controllers for LL assistance are LDA, SVM, GMM, and ANN (Delorme et al., 2010, 2011).

#### Linear discriminant analysis

One of the most extensive and successfully deployed classification algorithms, in EEG-BCI for assistive technologies is the linear discriminant analysis (LDA). The method employs discriminant hyper-plane(s) in order to separate data representing two or more classes. Since it has low computational requirements, it is most suitable for online BCI systems. A feature *a* can be projected onto a direction defined by a unit vector $\hat{\omega }$, resulting in a scalar projection *b*, given by Equation 4:

(4) $$\begin{array}{r} b=\overset{⇀}{a}\;\cdot \;{\hat{\text{ω}}}^{2} \end{array}$$

The aim of LDA classification is to find a direction $\hat{\omega }$, such that, when projecting the data onto $\hat{\omega }$ it maximizes the distance between the means and minimizes the variance of the two classes (dimensionality reduction). It assumes a normal data distribution along with an equal covariance matrix for both classes (Lotte et al., 2007). LDA minimizes the expression given by Equation 5:

(5) $$\begin{array}{r} \frac{{\left(m_{\phi }-m_{\text{Ψ}}\right)}^{2}}{s_{\phi }^{2}+s_{\text{Ψ}}^{2}} \end{array}$$

where *m*_ϕ_ and *m*_Ψ_ are the means and *s*_ϕ_ and *s*_Ψ_ are the standard deviations of the two respective classes, after projecting the features onto $\hat{\omega }$. EEG-BCI lower exoskeletons used LDA for the reduction of data dimensionality (Kilicarslan et al., 2013). EEG-BCI lower orthosis employed a 12-fold LDA using voting rule for decision making in selection of speed (Lotte et al., 2009; Duvinage et al., 2012). Dimensionality reduction, using CPCA and approximate information discriminant analysis (AIDA), were used in the robotic gait orthosis system (Do et al., 2011, 2013). The BCI-driven orthosis (Xu et al., 2014) used the manifold based non-linear dimensionality reduction method, called locality preserving projection (LPP), along with LDA, to detect MRCPs. EEG-BCI wheelchairs successfully deployed LDA (Galán et al., 2008; Iturrate et al., 2009a,b). LDA was successfully used for translation of EEG signal into movement commands in humanoids (Chae et al., 2011a,b, 2012).

#### Support vector machine

The goal of SVM classifier is to maximize the distance between the separating hyper plane and the nearest training point(s) also termed support vectors. The separating hyper plane in the 2D feature space is given by the Equation 6:

(6) $$\begin{array}{r} y={\text{ω}}^{T}x+b \end{array}$$

where ω, *x* ∈ *R*^2^ and *b* ∈ *R*^1^. The hyper plane (also called the decision border) divides the feature space into two parts. Classified results depend on which side of the hyper plane the example is located. In SVM, the distances between a hyper plane and the nearest examples are called margins.

Though SVM is a linear classifier, it can be made with non-linear decision boundaries using non-linear kernel functions, such as Gaussian or radial basis functions (known as RBF). The non-linear SVM offers a more flexible decision boundary, resulting in an increase in classification accuracy. The kernel functions, however, could be computationally more demanding. EEG-BCI wheelchairs have successfully used linear SVM for dynamic feature classification (Bell et al., 2008; Choi and Cichocki, 2008; Ferreira et al., 2008; Rebsamen et al., 2010; Belluomo et al., 2011). It was also successfully implemented in EEG-BCI humanoid (Bell et al., 2008) and mobile robots (Ferreira et al., 2008; Belluomo et al., 2011).

#### Gaussian mixture model

The GMM is an unsupervised classifier. This implies that the training samples of a classifier are not labeled to show their class. More precisely, what makes GMM unsupervised is that during the training of the classifier, estimation is done for the underlying probability density functions of the observations (Scherrer, 2007). Several EEG-BCI applications utilized the GMM as a feature classifier, such as lower exoskeletons, wheelchairs and mobile robots (Galán et al., 2008; Millán et al., 2009; Carlson and Millan, 2013; Kilicarslan et al., 2013).

#### Artificial neural network

The ANNs are non-linear classifiers inspired by human's nervous system ability to adaptively react to changes in surroundings. They are commonly used in pattern recognition problems, due to their post-training capability to recognize sets of training-data-related patterns. ANNs comprise of assemblies of artificial neurons that allow the drawing of non-linear decision boundaries. They can be used in different algorithms including multilayer perception, Gaussian classifier, learning vector quantization, RBF neural networks, etc. (Anthony and Bartlett, 2009). In their proposed model for lower exoskeleton (Gancet et al., 2011, 2012), they aim at adopting processing method as dynamic recurrent neural network (DRNN).

### Execution layer

Once classified, the desired state of user intent is carried to the execution layer for an error approximation. The approximation in reference to the present state of the device is used to drive the actuator for reducing any error. The execution layer of control is highly device-specific. It could rely on feedforward or feedback loops (Tucker et al., 2015).

Feedforward control needs some model to predict the system's future state, based on the past and present set of inputs and the device state. Aforementioned control inputs can be effective for reducing the undesired interaction forces, that could occur due to the added mass, inertia and friction of the device (Murray and Goldfarb, 2012). On the contrary feedback controllers do not require a model of the system, but require an estimate of the current state. The controller compares current state with the desired state of the device and modulates the power input to the device accordingly (Millán et al., 2009; Duvinage et al., 2012; Noda et al., 2012; Contreras-Vidal and Grossman, 2013; Do et al., 2013; Kilicarslan et al., 2013; Xu et al., 2014; Contreras-Vidal et al., 2016).

## Shared control

Shared control is used to couple the user's intelligence, i.e., cognitive signals with precise capabilities of the robotic device given the context of surroundings, resulting in reduced workload for the user to continuously deliver commands to drive the robotic device. Inputs to the shared control module are sensory readings of the robotic device and output of the BCI operator (classified signal). The classified signal is combined with the robot's precise parameter e.g., velocity to generate smoother driving output. Several assistive technologies for motor impairment have successfully employed shared controllers for navigational assistance to maneuver the assistive devices in different directions, independently and safely (Galán et al., 2008; Millán et al., 2009; Tonin et al., 2010, 2011; Carlson and Millan, 2013).

This refers to the idea of switching between operators, i.e., if the user needs no navigational assistance he will be granted full control over the robotic device; otherwise, sole mental commands will be used and modified by the system. One key aspect of shared control is the two-way communication between the human and the robot. The shared control is beneficial primarily for navigational directions. In the case of robots with only three possible steering mental commands such as forward, left, and right, there is a need of assistance by the device for fine maneuvering. Secondly, the cognitive commands might not always be perfect, i.e., could be vague. In the case of errors, an extra navigational safety is required by the system to interpret the meaning of the command. In this way the system would be able to perceive any new environment.

## Lower-limb assistive-robot applications in different environments

The last integral part, of the control framework, is the robotic device, as observed in Figure 2. In this section, the current state-of-the-art EEG-based activity mode recognition in a BCI for control of LL assistive devices is summarized in Table 1.

**Table 1:** Key features of EEG-based activity mode recognition exoskeletons, orthosis, wheelchairs and assistive robots for rehabilitation.

**Devices**	**Brain activity**	**Pre-processing and feature extraction**	**Classifier**	**Classifi-cation accuracy (%)**	**Key findings**	**Type of support and applications**	**References**
NeuroRex	Oscillatory rhythms	Bandpass filter, PSD analysis	GMM, LDA	-, >90 (GMM), -	For standing-up, self-balancing, walking and backing, turning, ascending and descending stairs applications. An augmented form of Locomotor Therapy (LT)	Lower body exoskeleton based on user intent control for walking independently for subjects with paraparesis, complete paraplegia, stroke and SCI	Noda et al., 2012; Contreras-Vidal and Grossman, 2013; Kilicarslan et al., 2013
MIND-WALK-ER	SSVEP^*^	ICA	DRNN Chéron et al., 2011, KNN	-, -, 92.6% (online)	Exploitation of motor cortex EEG signals for generating online legs kinematics angles corresponding to walking pattern and pace as imagined by user deploying VR	Crutch-less assistive LL exoskeleton for walk empowering (dynamic balance) for SCI patients with intact brain capabilities	Gancet et al., 2011, 2012; Kwak et al., 2015
HAL® Exo-skeleton	SEP	Bandpass filter	-		Significant improvement in paired-pulse SEP in SCI patients compared to the controls at baseline following training. The robotic-assisted BWSTT in SCI patients is capable of inducing cortical plasticity following highly repetitive, active locomotive use of paretic legs.	HAL® exoskeleton-assisted bodyweight supported treadmill training (BWSTT) for improving walking function in SCI patients	Sczesny-Kaiser et al., 2015
Five-State Foot Lifter	P300^*^	Temporal high-pass filter, xDAWN-based spatial filter Rivet et al., 2009, epoch averaging, SFFS	LDA (using voting rule for decision making)	83 ± 15.5% (walking) 75% (walking)	Proof of the concept of combining a human gait model based on CPG widely used in robotics and P300 based BCI to consider user's intent. This CPG allowed to automatically generate a periodic gait pattern/behavior of the patient and his desired speed. No required training by the user to manage the P300 paradigm provided by augmented reality eyewear for external stimulus presentation.	A five-state foot lifter orthosis for sitting, standing and walking at four speeds & a non-control state for stroke patients unable to lift their feet or foot drop problems Pilot study for ambulatory BCI	Lotte et al., 2009; Duvinage et al., 2012
BCI-RoGO	Oscillatory rhythms^**^	FFT, PSD, CPCA	AIDA, linear Bayesian classifier	>85%, -, -	Development of EEG prediction model based on idling and KMI states. Preliminary evidence from results reflect the feasibility of restoring brain-controlled walking after SCI.	BCI Robotic gait orthosis for SCI, tetraplegia, and paraplegia patients to improve neurological outcomes beyond those of standard therapy to improve ambulation	Wang et al., 2010; Do et al., 2011, 2013
BCI-MAFO	MRCP^***^	Bandpass filter, large Laplacian filter, ANOVA	LPP and LDA	73 ± 10.3%.	Efficient induction of cortical neuroplasticity in healthy subjects with a short intervention procedure to use self-paced BCI for binary control of the robotic orthosis.	BCI-driven motorized ankle-foot orthosis (MAFO). An ambulatory rehabilitation-tool for stroke patients	Xu et al., 2014
BCI Wheel-chair	Oscillatory rhythms	Spatial filter (CAR), Laplacian filter, PSD (Welch method), CVA, Bandpass filter, FFT	Gaussian model, LDA	≥90%, -, ≥80%, 80%	Reduced cognitive workload due to BCI protocol coupled with shared control, compared to previous systems. Spontaneous control given to user to move left, right or forward and avoid obstacles automatically by perceiving surrounding environment, no waiting for external cues compared to synchronous P300 protocol. Based on combination of cheaper sensors for providing controller with environmental feedback.	Brain-actuated wheelchair for users with severe mobility impairment. Suitable for experienced/inexperienced users to continuously and safely operate with even complex navigation independently	Vanacker et al., 2007; Galán et al., 2008; Millán et al., 2009; Carlson and Millan, 2013
P300 BCI Wheel-chair	P300, ERP	Bandpass filter, moving average filter	SVM, Gaussian model, LDA	≈100%, ≈100%, ≥94%, ≥94%, 100%, 100%, ≥95%, ≥85.8%	Successfully targeted people suffering from a very low information transfer rate using the P300 paradigm, using virtual guiding paths and predictable trajectories. Incorporation of *mu/beta* (a faster BCI) to stop wheelchair. Provision of destination selection from predefined localities in the menu.	BCI wheelchair for locked-in or ALS patients. Intelligent and safe BCI wheelchair where known surroundings as, toilet, kitchen, bedroom and living room in house is highlighted by standard oddball paradigm.	Rebsamen et al., 2007, 2010; Pires et al., 2008; Iturrate et al., 2009a,b; Palankar et al., 2009; Lopes et al., 2011; Kaufmann et al., 2014
BMI wheel-chair	Oscillatory rhythms^*^	2nd order BSS with AMUSE algorithm, CSP filter, Bandpass filter	SVM	-	Effective feedback training method resulting in multi DOFs/freely controlling wheelchair parallel to controlling with a joystick	BCI wheelchair based on MI protocol for motor impaired patients.	Choi and Cichocki, 2008
BCI mobile robot/humanoid	Oscillatory rhythms	Bandpass filter, Laplacian filter, PSD (Welch method)	Statistical Gaussian model	74%, ≥75.6%, 81%, ≥75.6%, -, -	Allow subjects to complete complex tasks in same time and with same number of commands as required by manual control	BCI based telepresence robot for left/right steering via imagination of left/ right hand or feet movement of physically impaired people. Control navigation of humanoid robot via MI.	Millan et al., 2004; Tonin et al., 2010, 2011; Chae et al., 2011a,b, 2012
BCI mobile robot/humanoid	SMR, ERP, P300^*^	Spatial filter, temporal filter, Bandpass filter	SVM	95%, -, 95%, ≥93%, 80.5%	Development of an interactive BCI system to control twin coordinated mobile robot movements via two EEG signals (imagery left-right arm). The concentration and relaxation states of visual cortex, was used to allow operator to successfully control a robot without using hands. Successful control of BCI humanoid for sophisticated interaction with the environment, involving not only navigation but also manipulation and transport of objects.	BCI controlled mobile and telepresence robots for navigation in required direction for motor disability assistance. BCI controlled humanoid for navigation assistance as well as transportation of objects.	Bell et al., 2008; Ferreira et al., 2008; Belluomo et al., 2011; Escolano et al., 2012; De Venuto et al., 2017

* They used combined EEG and EMG modalities in their system.

** *They used combined EEG, FES, and EMG modalities in their BCI orthosis*.

*** *They used combined EEG and TMS modalities for brain signal acquisition and for classification purposes, they used additional features from EMG in their BCI orthosis*.

### BCI exoskeletons

In order to control a LL robotic exoskeleton (NeuroRex), Contreras-Vidal and Grossman (2013) and Kilicarslan et al. (2013) decoded neural data for human walking from Presacco et al. (2011b). They evaluated the degree of cognitive-motor-body adaptations while using portable robot. Their results proved that NeuroRex can be regarded as an augmented system of locomotor therapy (LT) by reviewing its initial validation in a paraplegic patient having SCI. They also performed comprehensive clinic assessments for user safety protection.

The MINDWALKER (Gancet et al., 2011, 2012) is another project where researchers proposed a novel idea of presenting the SCI patients with intact brain capabilities. The facility of crutch-less assistive LL exoskeleton is based on brain neural-computer interface (BNCI) control for balanced walking patterns. It also evaluated the potential effects of Virtual Reality (VR) based technology that could support patient/user training for reaching a high confidence level for controlling the exoskeleton virtually before the real transition. Other brain controlled exoskeletons are reported in Noda et al. (2012), Kwak et al. (2015), Sczesny-Kaiser et al. (2015), and Lee et al. (2017).

### BCI orthosis

EEG-based activity mode recognition for orthotic devices has been investigated by Duvinage et al. (2012). They proved the concept of considering user's intent by combining CPG-based human gait model and classic P300-BCI for five different states; three speed variations, a stop state and a non-control state. Using unnatural P300 command by augmented reality eyewear (from Vuzix, Rchester, USA) decision was sent to the Virtual Reality Peripheral Network (VRPN) server to be exploited while wearing LL orthosis. This was based on the pilot study carried by Lotte et al. (2009), where a solution to the constraints, such as deterioration of signals (during ambulation), was avoided by using slow P300 for control during sitting, walking and standing. Authors of Castermans et al. (2011a) used an experimental protocol to limit movement artifacts present in EEG signals compared to real walk on treadmill. They suggested that rhythmic EEG activity could be exploited for driving a user intent-based foot-ankle orthosis built on PCPG algorithm. Similar investigation was conducted by Raethjen et al. (2008).

In their work, Do et al. (2013) proposed a novel approach of BCI controlled lower extremity orthotics to restore LL ambulation for partially and complete SCI subjects suffering from cardiovascular disease, osteoporosis, metabolic derangements and pressure ulcers. They developed an EEG prediction model to operate the BCI online and tested the commercial robotic gait orthosis system (RoGO) for two states, idling and walking KMI. Similarly, testing for intuitive and self-paced control of ambulation was also done with an avatar in a virtual reality environment (VRE) (Wang et al., 2012; King et al., 2013). Other similar investigations are reported in Wang et al. (2010) and Do et al. (2011).

The BCI driven motorized ankle-foot orthoses, known as (BCI-MAFO), intended for stroke rehabilitation was presented in Xu et al. (2014). Their system was able to detect imaginary dorsiflexion movements (for walking gait) within a short latency, by analyzing MRCPs. Upon each detection, the MAFO was triggered to elicit passive dorsiflexion, hence, providing the user a binary control of robotic orthosis. The MEP was elicited by transcranial magnetic stimulation (TMS); the results reflected an effective way to induce cortical plasticity for motor function rehabilitation.

### BCI wheelchairs, humanoids, and mobile robots

Assistive technologies such as wheelchairs controlled via EEG-BCI have extensively been researched. In their work, Carlson and Millan (2013) proposed the idea of combining a commercial wheelchair and BCI with a shared control protocol. The paradigm was based on KMI of left/right hand, both feet, or in idle state; each against three distinct tasks as move left/right or forward by avoiding obstacles. Modifications in the commercial mid-wheel drive model (by Invacare Corporation) were directly controlled by a laptop. An interface module, based on remote joystick, was used between the laptop and wheelchair's CANBUS-based control network. Wheel-encoders were added for motion feedback alongside sonar sensors and webcams for environment feedback to the controller using cheap sensors compared to other systems. Previous solution required continuous commands from the user, in order to drive the wheelchair, that ended up in high user workload (Millán et al., 2009). Other similar systems were proposed by Vanacker et al. (2007) and Galán et al. (2008).

Research on the challenges faced during fully control automated wheelchairs with BCI was done by Rebsamen et al. (2007, 2010). Their results proved that if synchronous evoked P300 signals are used for mobile commands, and oscillatory rhythms are used for stop command, the system is efficient and safe enough to drive the real-time wheelchair in possible directions. They used Yamaha JW-I power wheelchair with two optical rotary encoders attached to glide-wheels for odometry, a bar code scanner for global positioning and a proximity sensor mounted in front of the wheelchair for collision avoidance. User could reach the destination, by selecting amongst a list of pre-defined locations. This was primarily for patients with lost voluntary muscle control, but intact cognitive behavior who could use a BCI, such as LL amputees.

Other P300-BCI wheelchairs' research include work done by Iturrate et al. (2009a,b) where the system relied on synchronous stimulus-driven protocol. The work done by Palankar et al. (2009) focused on, completely and partially locked-in patients, and provided them with an effective model of a 9-DOF wheelchair-mounted robotic arm (WMRA) system. Pires et al. (2008) and Lopes et al. (2011) contributed in visual P300 based BCI for steering wheelchair assisted by shared-control. Kaufmann et al. (2014) validated the feasibility of a BCI based on tactually-evoked ERP for wheelchair control. Other wheelchairs controlled via EEG-based BCI include (Choi and Cichocki, 2008; Tsui et al., 2011; Huang et al., 2012; De Venuto et al., 2017).

In their report (Tonin et al., 2010, 2011) presented a BMI-controlled telepresence robot for people with motor impairment that could allow them completion of complex tasks, in similar time as that consumed by healthy subjects. They were able to steer Robotino^TM^ (by FESTO), via asynchronous KMI of left/right hand and feet. The system incorporated shared control for obstacle avoidance, safety measures and for interpreting user intentions to reach goal autonomously. A similar project was earlier presented by Millan et al. (2004) for mobile robot control in indoor environment via EEG. In order to recognize environment situations, a multilayer perception was implemented. Sensory readings were mapped to 6 classes of environmental states: forward movement, turn left, follow left wall, right turn, follow right wall and stop. These environmental states were generated against mental tasks as relax, KMI of left/right hand, cube rotation imagery, subtraction and word association. Research for control of two coordinated mobile robots, via SMR and ERP, that could be useful for motor impaired people, is done by Belluomo et al. (2011). Similarly mobile robot (Pioneer 2-DX) control based on *mu* ERD/ERS was done by Ferreira et al. (2008).

As per our knowledge, reflected from the literature, there is no viable active prosthetic ankle-foot, or prosthetic LL device, controlled via EEG-BCI for amputees.

## Practical challenges

In order to design a controller for an assistive-robot device there is a need of a seamless integration between the BCI operator, and the execution of required tasks from the output device with minimal cognitive disruption. However, there are challenges associated to the real-time implementation of the system, when dealt with motor impaired population. Some open problems and challenges associated to wearable systems have recently been summarized in (Deng et al., 2018; Lazarou et al., 2018; Semprini et al., 2018). The following sections discuss in detail practical challenges associated to EEG-BCI wearable and assistive technologies.

### Wearable lower-limb device challenges

A critical need for reliable EEG-BCI is required that could interpret user intent and make context-based decisions from the user's present internal state. This would allow a direct and voluntary operation of the wearable LL devices beyond the user's affected physical, cognitive or sensory capabilities. With wearable LL devices it is observed that they did not embed shared controllers. The system should involve the development of reliable discrete classifiers, combined with continuous (model-based) neural interfaces, to predict the subject's intent without needing continuous supervisory control, but an “*assist-as-needed”* control from the BCI. Wearable LL technologies should embed features such as, self-calibration, self-analysis (with backward-forward failure attribution analysis) and error-correction. This is followed by adopting appropriate behavioral testing methods for performance evaluations of the system.

Clinical evaluation of wearables needs standardized safety and tolerability assessment of important factors such as cardiometabolic, musculoskeletal, skin, and biomechanical risks, followed by the assessment of cognitive-behavioral discrepancies that define the user profile. Cardiorespiratory safety is of principal importance as individuals with stroke and SCI may have autonomic instability that can alter the pressure of blood-flow. Their heart rates may not respond correctly to increased cardiorespiratory demands, depending on the lesion intensity. The cardiorespiratory demands of supported BCI-exoskeleton/orthosis usage must primarily be assessed and carefully monitored also for reasons as: (1) the mean peak heart fitness levels after SCI vary considerably depending on the lesion characteristics, but are generally much lower than normal; and (2) the skeletal muscle after SCI (or any central-nervous system injury) shifts in a shortfall severity from slow to a fast jerk molecular composition. Patients with abnormal gait biomechanics and fitness levels must show adequate cardiorespiratory tolerance based on subject perceived exertion scales, and objective monitoring of metabolic profiles. This metabolic surveillance, along with careful clinical measures, to assess muscle injury, is inevitable for validating the cardiorespiratory, metabolic, and muscle safety during exoskeleton/orthosis use.

During rehabilitation, the wearable robotics may impose unusual joint kinetics and kinematics that could potentially injure bone or skin, particularly in stroke or SCI patients that usually have osteoporosis, unusual spasticity patterns, or contractures. For safe utilization a standard screening for assessment of bone health using dual X-ray absorptiometry and identification of abnormal torque or impulses ahead of time, could retain from injury. There should be a careful consideration between engineers, clinicians, and subjects with neurological disability to rightly apply this new technology.

Substantial research and understanding of the cortical representations, for the perception of bipedal locomotion, is vital for evaluating changes in cortical dynamics when wearing closed-loop BCI portable devices, and gauging on how these changes are correlated with gait adaptation. As the BCI wearable devices are designed to be stable, they have to finish one complete cycle of gait before stopping, resulting in a slow time-response compared to the model's output. This is why in some systems the subject has to keep standing, as long as he can, after stopping the robot for continuously recording the model's output state.

With P300-wearable LL devices, the decision time is relatively slow for real-time applications such as walking. The solution could involve implementation of more complex pipelines that include artifact removal techniques specific to gait-artifacts, followed by a better management of stimulus presentation duration. The P300 pipeline does not allow working asynchronously, which is an important aspect for the patient's comfort (can be tiring). Following this, the poor experimental paradigm that usually includes a screen on a treadmill is not applicable for street walking; accordingly, an augmented reality eyewear seems to be indispensable.

### Assistive-robot challenges

Clinical evaluations revealed that subjects with poor BCI performance require an extra need for assistance while maneuvering assistive-robots during complex path plans such as narrow corridors, despite the arduous BCI training.

The use of adaptive assistance with BCI wheelchairs increases the task performance of the user; however, the fixed activation levels of the system do not integrate the user's performance. This is due to the varying fatigue and hormone levels of the user, due to which the shared controller may not offer constant level of assistance. Consequently, similar system behavior is always activated when the activation threshold is reached, even though an experienced user might still be able to recover from the disorientation on its own. System performance could be increased, if a user model is built at runtime, and the level of experience to determine the thresholds is estimated when the system behavior is activated.

Various customized filtering approaches have been deployed by researchers during different states of wheelchair use, for instance, the regular on and off switching of filter in between sessions of start and stop. Given in Kwak et al. (2015), when the filter was switched on or off, the subject was required to use another mental mode (or at least adapt its existing one) as the driving system was different when the filtering was applied. This resulted in a confusion mode which is a common problem in shared control systems. When the subject's acquired strategies are built up using one driving system (i.e., without filtering) and applied to the other situation (i.e., with filtering), it ends up in a weak performance, leading to a situation where the environmental filter is actually working against the user's intention. With present BCI-wheelchairs that incorporate shared controllers, if the activation levels of the system do not integrate the user's performance, it could lead to degradation or loss of function.

Reportedly P300-wheelchairs were too slow to stop in real-time, after the selection of a sub-goal from menu, the user has to focus on a validation option, due to which the wheelchair stops and waits for the next command (followed by validation) from the user. Consequently this ends up in more stationary positions than actually moving to specific destinations.

## Conclusions

In this paper, we have presented a comprehensive review of the state-of-the-art EEG-BCI controlled wearable and assistive technologies for users having neuromotor disorder, SCI, stroke, disarticulation or amputation of residual LL. All reviewed applications are presented in the form of a generalized BCI control framework. The control framework is inclusive of the user, the BCI operator, the shared controller, and the robot device with the environment. Each element of the control framework was discussed in detail. The BCI operator is based on sub-layers, each of which is highlighting the feature extraction, classification and execution methods respectively, employed by each application. The reviewed applications comprised of oscillatory rhythms, event-related and evoked potentials as input signals. The EEG-BCI based portable and assistive device applications included exoskeletons, orthosis, wheelchairs, mobile/navigation robots and humanoids. Key features from each application were discussed and presented in the Table 1.

Based on the review we concluded that LL tasks, such as knee, or hip joint movements, have never been explicitly employed as MI or ME tasks in any BCI experimental protocol. Only foot or upper limb kinesthetic tasks are deployed. Additionally, it is observed that the EEG-based activity mode recognition, used to control wearable LL devices, only comprise of exoskeletons and orthosis. No viable prosthetic ankle-foot, or prosthetic LL device, employing EEG signals, for activity mode recognition, is currently available.

In most applications based on P300, strong output signals were observed that resulted in accurate command functions. It was followed by a slow performance pace and a loss in the user concentration due to stimulus presentation. On the contrary, applications employing SMR, where no stimulus protocol is involved, reflected a faster performance speed, followed by a weaker output signal during asynchronous mode.

Performance of EEG-based BCI, deployed by assistive technologies, is constrained due to the design of non-invasive modalities, compared to invasive ones and due to the limited size of features employed. In the case of complex movements more sets of parameters are required to execute a seamless output. This is still one of the challenging problems that require expertise to develop efficient and robust algorithms to apprehend user's motion intention.

In the most of the reviewed applications, there is a lack of quantitative performance indicators for the algorithms' evaluations. There is no explicit signal classification, percentage given. Error measurements between expected and real system trajectories are missing. There is no indication about the measurements of the user-energy consumption, the walking endurance and the system costs. Finally, an important issue of carrying tests under realistic conditions, with patients having LL pathologies, needs special attention, provided the observations make the comparison of the dynamic behavior of each application difficult.

## Author contributions

MT devised, drafted, structured, analyzed, and coordinated reading and writing of this review. She contributed text throughout, generated the figures and developed the structure of the generalized control framework and provided final approval of the manuscript. PT contributed to analysis, critical revision, provided feedback and final approval on the manuscript. MS contributed to the Figure 1, analyzed, critically revised, provided feedback and final approval on the manuscript. All authors read and approved the final version of the manuscript. All authors agree to be accountable for all aspects of the work in ensuring that questions related to the accuracy or integrity of any part of the work are appropriately investigated and resolved.

### Conflict of interest statement

The authors declare that the research was conducted in the absence of any commercial or financial relationships that could be construed as a potential conflict of interest.
